# Intracellular *Propionibacterium acnes* Infection in Glandular Epithelium and Stromal Macrophages of the Prostate with or without Cancer

**DOI:** 10.1371/journal.pone.0090324

**Published:** 2014-02-28

**Authors:** Yuan Bae, Takashi Ito, Tadatsune Iida, Keisuke Uchida, Masaki Sekine, Yutaka Nakajima, Jiro Kumagai, Tetsuji Yokoyama, Hiroshi Kawachi, Takumi Akashi, Yoshinobu Eishi

**Affiliations:** 1 Department of Human Pathology, Tokyo Medical and Dental University Graduate School, Bunkyo-ku, Tokyo, Japan; 2 Division of Surgical Pathology, Tokyo Medical and Dental University Graduate School, Bunkyo-ku, Tokyo, Japan; 3 Department of Pathology, Yokohama City Minato Red Cross Hospital, Yokohama, Kanagawa, Japan; 4 Department of Health Promotion, National Institute of Public Health, Wako, Saitama, Japan; Oklahoma University Health Sciences Center, United States of America

## Abstract

**Background:**

Recent reports on *Propionibacterium acnes* (*P. acnes*) suggest that this bacterium is prevalent in the prostate, is associated with acute and chronic prostatic inflammation, and might have a role in prostate carcinogenesis.

**Methods:**

To evaluate the pathogenic role of this indigenous bacterium, we screened for the bacterium in radical prostatectomy specimens using enzyme immunohistochemistry with a novel *P. acnes*-specific monoclonal antibody (PAL antibody), together with an anti-nuclear factor-kappa B (NF-κB) antibody. We examined formalin-fixed and paraffin-embedded tissue sections of radical prostatectomy specimens from 28 patients with prostate cancer and 18 age-matched control patients with bladder cancer, but without prostate cancer.

**Results:**

Immunohistochemistry with the PAL antibody revealed small round bodies within some non-cancerous glandular epithelium and stromal macrophages in most prostate samples. Prostate cancer samples had higher frequencies of either cytoplasmic *P. acnes* or nuclear NF-κB expression of glandular epithelium and higher numbers of stromal macrophages with *P. acnes* than control samples. These parameters were also higher in the peripheral zone than in the transitional zone of the prostate, especially in prostate cancer samples. Nuclear NF-κB expression was more frequent in glands with *P. acnes* than in glands without *P. acnes*. The number of stromal macrophages with the bacterium correlated with the grade of chronic inflammation in both the PZ and TZ areas and with the grade of acute inflammation in the TZ area.

**Conclusions:**

Immunohistochemical analysis with a novel monoclonal antibody for detecting *P. acnes* in the prostate suggested that intraepithelial *P. acnes* infection in non-cancerous prostate glands and inflammation caused by the bacterium may contribute to the development of prostate cancer.

## Introduction

Prostate cancer is the second most common cancer in men worldwide, with an estimated 900,000 cases and 258,000 deaths in 2008 [1]. While several risk factors for prostate cancer have been identified, such as ethnic origin, age, family history, and diet [2], the exact etiology of prostate cancer remains unknown. Many recent studies provide evidence that chronic inflammation is an important contributing factor for prostate carcinogenesis by causing DNA damage, promoting cellular turnover, and creating a tissue microenvironment that enhances cell replication, migration, and angiogenesis [3], [4].

In the inflammatory response, transcriptional factors such as nuclear factor-kappaB (NF-κB) are activated upon binding of pattern-recognition receptors (Toll-like receptors, nucleotide-binding oligomerization domain [NOD]-like receptors, and others), proinflammatory cytokine receptors (tumor necrosis factor-α or interleukin [IL]-1), and antigen receptors [5]–[8]. Although NF-κB does not fit the classical definition of an oncogene, it is a powerful activator of the malignant state and regulates the expression of target genes important for cell proliferation, survival, angiogenesis, and tissue repair [8]–[12].

Exposure to environmental factors, such as infectious agents, dietary carcinogens, and hormonal imbalances, is thought to lead to injury of the prostate and the development of chronic inflammation [3]. Recent reports showed that *Propionibacterium acnes* (*P. acnes*) is frequently detected in prostate tissue from patients with prostatitis and prostate cancer, and that the bacterium is associated with acute and chronic prostatic inflammation and might have a role in prostate carcinogenesis [13]–[21].

*P. acnes* is a Gram-positive, non-spore-forming, anaerobic bacillus found predominantly in the sebaceous gland-rich areas of the skin in adults [22]. The indigenous bacterium is also isolated from the conjunctiva, mouth, and intestine [23]. Historically, *P. acnes* was thought to be of low virulence, but was recently found to be the causative agent in various pathologies. *P. acnes* is most notably implicated in acne vulgaris [24], but the bacterium might also be associated with a number of inflammatory conditions, such as endocarditis, joint and central nervous infections, and sarcoidosis [25]–[28].

We previously reported that many (71%) serotype I clinical isolates of *P. acnes* invade epithelial cells [29], and intraepithelial *P. acnes* infection activates NF-κB in both a NOD1- and NOD2-dependent manner [30]. Despite accumulating evidence of *P. acnes* infection in the prostate by bacterial culture or polymerase chain reaction methods, there are only a few reports in which the bacterium was located in prostate tissues by in situ immunofluorescence methods with a polyclonal antibody to *P. acnes* [31] or multicolor fluorescence in situ hybridization methods targeting *P. acnes* 23S rRNA [14].

To further investigate the etiologic association between *P. acnes* and inflammation or carcinogenesis, not only the bacterium but also histologic features of the prostate tissue need to be analyzed in identical histologic sections. The aim of the present study was to locate *P. acnes* in prostate tissue under light microscopy by enzyme immunohistochemistry. For this purpose, we developed a novel anti-*P. acnes* monoclonal antibody that reacts with the bacteria in formalin-fixed and paraffin-embedded prostate tissue sections. To evaluate the pathogenic role of this indigenous bacterium in the development of prostate cancer, we examined radical prostatectomy samples obtained from patients with or without prostate cancer by immunohistochemistry with the novel antibody to *P. acnes* and an antibody to NF-κB, which was used to determine a possible correlation between *P. acnes* infection and nuclear NF-κB expression in prostate glands. Furthermore, we analyzed whether *P. acnes* infection status was associated with prostate cancer risk.

## Materials and Methods

### Ethics statement

This study was approved by the ethics committee of Tokyo Medical and Dental University (Registration No. 1373). Because the study involved immunostaining of clinically obtained and archived formalin-fixed and paraffin-embedded tissue specimen, the ethics committee approved waiver of specific informed consent in accordance with Ethical Guidelines for Clinical Studies (amended July 31, 2008) by Ministry of Health, Labour and Welfare of Japan. The animal experimental protocol used in this study was approved by the Center for Experimental Animal of Tokyo Medical and Dental University (Registration No. 0120203A) and was performed in accordance with the guidelines of the above center.

### Samples

We examined formalin-fixed and paraffin-embedded tissue sections of radical prostatectomy samples from 28 patients (age: 50–78 years) with prostate cancer who underwent surgery between 2008 and 2011 at the Tokyo Medical and Dental University Hospital, and from 18 control patients (age: 50–80 years) with bladder cancer, but without prostate cancer, who underwent surgery between 1994 and 2011 at the same hospital. Patients who received preoperative treatment were excluded from the study. The clinicopathologic profiles of the cases are shown in Table 1. Paraffin-embedded tissue blocks were selected from blocks prepared for routine pathologic examination. One horizontally cut section of the prostate including the verumontanum was identified in each case, and the samples were enrolled in the study when the cancer spread was limited to the right or left lobe in the section. One of the right and left lobes in a section free from cancer was analyzed to examine the distribution of *P. acnes* and the intraepithelial activation of NF-κB in non-cancerous tissue, and the other lobe was analyzed to detect *P. acnes* in cancer tissue.

**Table 1:** Clinicopathologic profiles of the cases.

Profiles	Value
Prostate cancer cases (n = 28)	
Age, yr, mean ± SD	66.4±6.7
PSA, ng/ml, mean ± SD	9.9±7.0
Gleason score	
6	3
7	19
8	4
9	2
Pathologic T stage	
pT2a	6
pT2b	3
pT2c	14
pT3a	4
pT3b	1
Control cases (n = 18)	
Age, yr, mean ± SD	67.3±9.7
Localization of the cancer	
Urinary bladder	17
Urinary bladder and renal pelvis	1
Histologic type of the cancer	
Urothelial carcinoma	15
Squamous cell carcinoma	2
Undifferentiated carcinoma	1
Pathologic T stage	
pTis	6
pTa	1
pT1	6
pT2	2
pT3	3

SD: standard deviations, PSA: prostate-specific antigen.

### Production of monoclonal antibody

A novel monoclonal antibody was developed to locate *P. acnes* in formalin-fixed and paraffin-embedded prostate tissue sections. The antibody was generated according to the protocol described in a laboratory manual [32] with modifications. BALB/c mice (CLEA Japan, Tokyo, Japan) were immunized with sonicated whole bacterial lysate of serotype I *P. acnes* isolated from human prostate in a previous study [29]. Hybridoma cell lines producing anti-*P. acnes* antibodies were checked by enzyme-linked immunosorbent assay with the bacterial antigens used as immunogens. Hybridoma cell lines with positive results were screened by immunostaining with formalin-fixed and paraffin-embedded tissue sections of *P. acnes*-injected rat liver. *P. acnes* injected rat liver was obtained by intravenous injection of 30 mg of heat-killed *P. acnes* into female Sprague-Dawley rats (CLEA Japan) 1 h before killing the rat. Similarly prepared liver tissue sections of rats injected by each strain of other control bacteria were also examined to confirm the specificity to *P. acnes*. Hybridoma cell lines producing the antibody that generated a strong reaction specific to *P. acnes* in rat liver sections were selected and further screened by immunostaining with formalin-fixed and paraffin-embedded prostate tissue sections of the specimen removed by prostatectomy in which a large number of *P. acnes* were cultured in the previous study [29]. The hybridoma producing the antibody that generated the most specific reaction product lacking any cross-reactivity to human prostate tissues, including lipofuscin pigments, was selected and cloned by two rounds of limiting dilution. A single hybridoma clone was then implanted into the intraperitoneal space of severe combined-immunodeficiency mice (CLEA Japan). At 1 or 2 weeks after implantation, ascites was collected and used as an undiluted antibody without further purification. The antibody was named PAL.

The specificity of the PAL antibody was examined by Western blot according to the previously described method [26] with serotype I *P. acnes* type strain (ATCC 6919 and ATCC 11827), serotype II *P. acnes* type strain (ATCC 11828), 19 clinical isolates of *P. acnes* (10 strains of serotype I and 9 strains of serotype II) from prostates [29], other cutaneous propionibacteria (*P. granulosum* ATCC 25564, *P. avidum*, ATCC 25577, and *P. lymphophilum* ATCC 27520), and other control bacteria (*Mycobacterium tuberculosis, Staphylococcus epidermidis, Escherichia coli*, *Bacteroides flagilis*, and *Enterrococcus faecalis*).

### Immunohistochemistry

Histologic sections (3 µm-thick) were cut from formalin-fixed and paraffin-embedded tissue samples and mounted on silane-coated slides (Muto Pure Chemicals, Tokyo, Japan). After the sections were de-paraffinized and rehydrated, they were microwaved (Microwave Processor H2850; Energy Beam Sciences, East Granby, CT) for 40 min at 97°C in 10 mmol/l citrate buffer (pH 6.0). The sections were then treated with 3% hydrogen peroxide in methanol for 10 min. The sections were first incubated with normal horse serum (Vectastain Universal Elite ABC Kit; Vector Laboratories, Burlingame, CA) and then incubated overnight at room temperature with either appropriately diluted PAL antibody (1∶60000) or anti-NF-κB antibody (1∶2000, EPITOMICS, Burlingame, CA), or a mixture of these two antibodies for cocktail immunostaining, in a humidified chamber. The sections were then incubated for 30 min with biotinylated secondary antibody, followed by 30 min incubation with streptavidin–peroxidase complex (Vectastain Universal Elite ABC Kit), both at room temperature. Before and after each step, the sections were washed in phosphate-buffered saline containing 0.5% Tween-20. The signal was developed as a brown reaction product using peroxidase substrate diaminobenzidine (HistofineSimplestain DAB Solution; Nichirei Bioscience Inc., Tokyo, Japan). All specimens were counterstained with Mayer's hematoxylin. Adjacent sections were also examined with hematoxylin and eosin staining for conventional histopathologic examination.

For simultaneous identification of cytoplasmic *P. acnes* and nuclear NF-κB expression in the same prostate tissue section, cocktail immunostaining with a mixture of PAL antibody and anti-NF-κB antibody as the primary antibody was performed in all specimens. To ensure the reliability of this method, two serial sections of identical specimens were examined by cocktail immunostaining and immunoenzyme double-staining, respectively. For the immunoenzyme double-staining, sections were first reacted with PAL antibody using avidin-biotin-complex method with the VECTASTAIN ABC-AP Kit (AK-5000, VECTOR) and the VECTOR Blue Alkaline Phosphatase Substrate Kit III (SK-5300, VECTOR), and then incubated for 5 min in denaturing solution (Denaturing solution kit, BRR001DH, Biocare Medical) and further incubated for 5 min in Dako REAL Peroxidase-Blocking Solution (S2023, Dako, Glostrup, Denmark). After these procedures, the sections underwent the secondary reaction with anti-NF-κB antibody, followed by immunohistochemistry using a polymer method with EnVision+ System-HRP Labelled Polymer (K4001, Dako) and HistofineSimplestain DAB Solution.

The same samples used for immunoenzyme double-staining were also analyzed by immunofluorescence double-staining to phenotype the cells with intracellular *P. acnes* using antibodies to epithelial cells and phagocytes; anti-cytokeratin monoclonal antibody (AE1/AE3, Dako) for epithelial cells, anti-human CD68 monoclonal antibody (clone KP1; Dako) for macrophages, or anti-human fascin monoclonal antibody (clone 55K-2; Dako) for dendritic cells, according to the previously described methods [33].

To confirm that the PAL antibody does not cross-react with lipofuscin pigments that are frequently found in prostatic glands, immunofluorescence staining with or without the PAL antibody was compared using serial prostate tissue sections, and immunoenzyme staining with the PAL antibody was followed by Fontana-Masson staining or fluorescence-microscopic observation for identical prostate tissue sections.

### Evaluation of immunohistochemical and histologic findings

To evaluate *P. acnes* infection and nuclear NF-κB expression in identical histologic sections, light-microscopic images of the sections immunostained with a mixture of PAL antibody and anti-NF-κB antibody (cocktail immunostaining) were incorporated into virtual slides with MIRAX MIDI BF/FL Digitizer for 12 Slides (Carl Zeiss MicroImaging, Jena, Germany) and examined using a Pannoramic Viewer 1.15 Service Pack 2 (3DHISTECH, Budapest, Hungary). Morphometric analysis of all prostate glands included in the sections was performed under high power view of the virtual slides. Each prostatic gland was considered *P. acnes*-positive when cytoplasmic positive signals by PAL antibody were observed in at least one epithelial cell of the gland. As for nuclear NF-κB expression, the gland was considered positive when the nucleus-positive signals were observed in at least one epithelial cell. Cytoplasmic NF-κB expression was not considered positive because activated NF-κB translocates from the cytoplasm to the nucleus [34], [35]. According to the presence or absence of cytoplasmic *P. acnes* and nuclear NF-κB expression for each gland, all prostate glands (total number of glands per section ranging from 933 to 5519 with a mean number of 2358) located in the areas of peripheral zone (PZ) or transitional zone (TZ) were categorized into four groups (*P. acnes*/NF-κB: +/+, +/−. −/+, and −/−), and glands categorized to each group were marked by a different color on the virtual slides. According to the results obtained by the four-group classification, the detection frequency of glands with either cytoplasmic *P. acnes* or nuclear NF-κB expression was calculated for each case in the PZ and TZ areas, respectively. All of the prostatic stromal cells with cytoplasmic signals by the PAL antibody were counted in the PZ and TZ areas, respectively, on the virtual slide sections that were immunostained with only PAL antibody. To evaluate the degree of acute and chronic inflammation, adjacent sections were examined with hematoxylin and eosin staining and classified into four grades (0, 1+, 2+, and 3+) according to the criteria used by Cohen et al. [13].

### Statistical analyses

The frequency (%) of *P.acnes*-positive glands or nuclear NF-κB-positive glands was calculated as the number of positive glands divided by the number of total glands for each patient; and the number of *P. acnes*-positive macrophages was counted for each patient. These parameters were summarized as median [25^th^ and 75^th^ percentiles] for each PZ and TZ area and compared between control and prostate cancer samples using a Mann-Whitney U test. The parameters in each control and prostate cancer sample were also compared between the PZ and TZ areas using the Wilcoxon singed-rank test. The association between these parameters and grade of acute or chronic inflammation was analyzed using the Spearman's rank correlation coefficient. Grade of acute or chronic inflammation was also compared between control and prostate cancer samples using a Mann-Whitney U test. The frequency of nuclear NF-κB expression in glands with *P. acnes* and glands without *P. acnes* was calculated for each patient, summarized as median [25^th^ and 75^th^ percentiles], and compared using the Wilcoxon singed-rank test in the PZ and TZ areas of control and prostate cancer samples. Spearman's rank correlation coefficient was used to evaluate the correlation between the frequency of *P.acnes*-positive glands and the number of *P. acnes*-positive stromal macrophages. The analyses were performed for the PZ and TZ areas, respectively. *p*-values were adjusted for multiple comparisons by Holm's method where appropriate. A *p*-value of less than 0.05 was considered to be statistically significant. StatView software (version 5.0; SAS Institute, Cary, NC) was used for all of the statistical calculations.

## Results

### Specificity of monoclonal antibody

The PAL antibody reacted with all strains of serotype I *P. acnes* and did not react with any strains of serotype II *P. acnes*, other cutaneous propionibacteria, or other control bacteria. The antibody recognized a *P. acnes*-specific epitope of the lipoteichoic acid commonly shared by all strains of serotype I *P. acnes*.

### Localization of *P. acnes* in prostate

**Figure 1:**
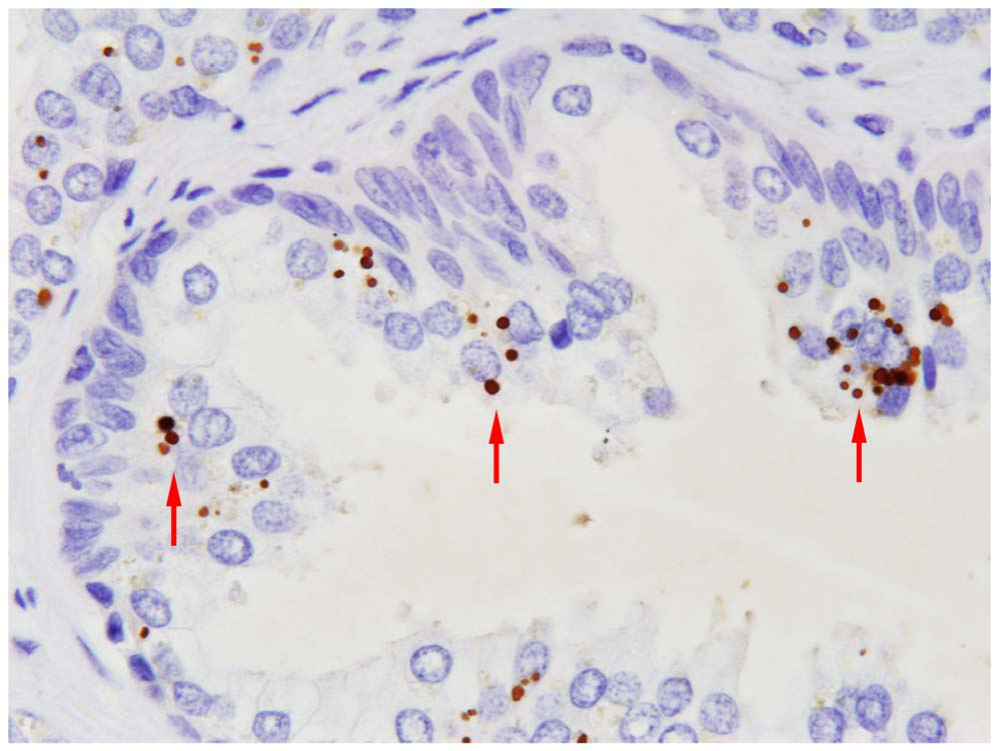
Immunohistochemistry of prostate glands with the PAL antibody. The antibody reacted with small round bodies (arrows) in some non-cancerous glandular epithelial cells.

**Figure 2:**
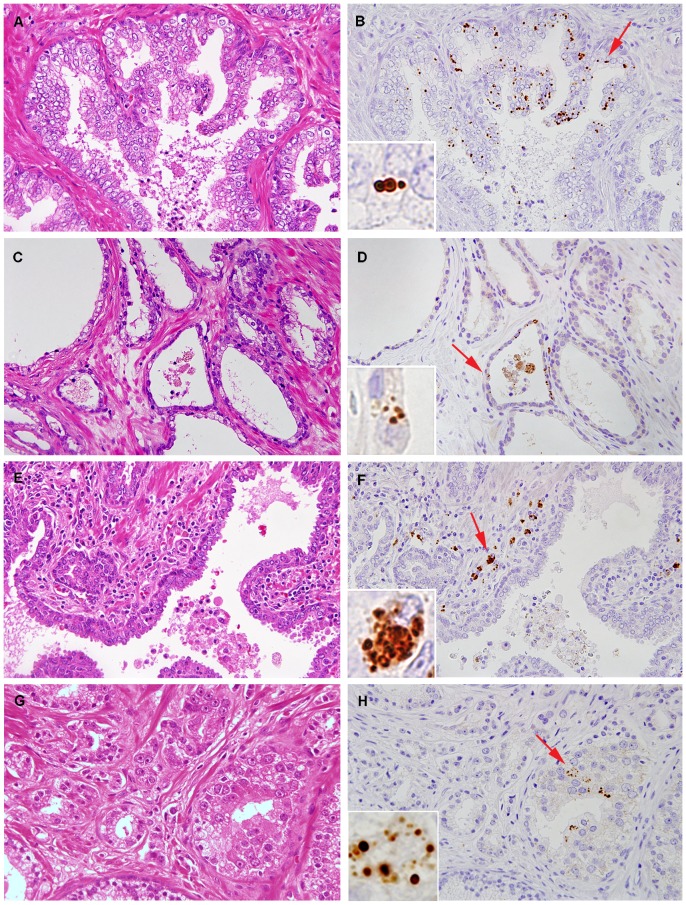
Immunohistochemistry of prostate tissues with the PAL antibody. Positive signals by the antibody were more frequent in glands with epithelial hyperplasia (A, B) than in normal or atrophic glands (C, D). In the prostatic stroma, positive signals were found in macrophage-like cells accompanied by mononuclear inflammatory cells (E, F). A few positive signals were infrequently found in some cancer cells (G, H). Higher magnification of the area indicated by the arrow is shown in the inset of each figure.

In the non-cancerous prostatic glands, the PAL antibody reacted with small round bodies in the epithelial cells (Figure 1). The small round bodies were observed in hyperplastic, normal, and atrophic epithelial cells, but more often in hyperplastic epithelial cells (Figure 2A–B) than in normal or atrophic cells (Figure 2C–D). In the prostatic stroma, PAL-positive small round bodies were also detected in macrophage-like cells, which were distributed sparsely in clusters in the stroma. These cells appeared in relatively high density near the atrophic glands, accompanied by mononuclear inflammatory cells (Figure 2E–F). In some foci of severe periglandular inflammation, macrophage-like PAL-positive cells infiltrated the glands and were sometimes detected in the luminal spaces.

**Figure 3:**
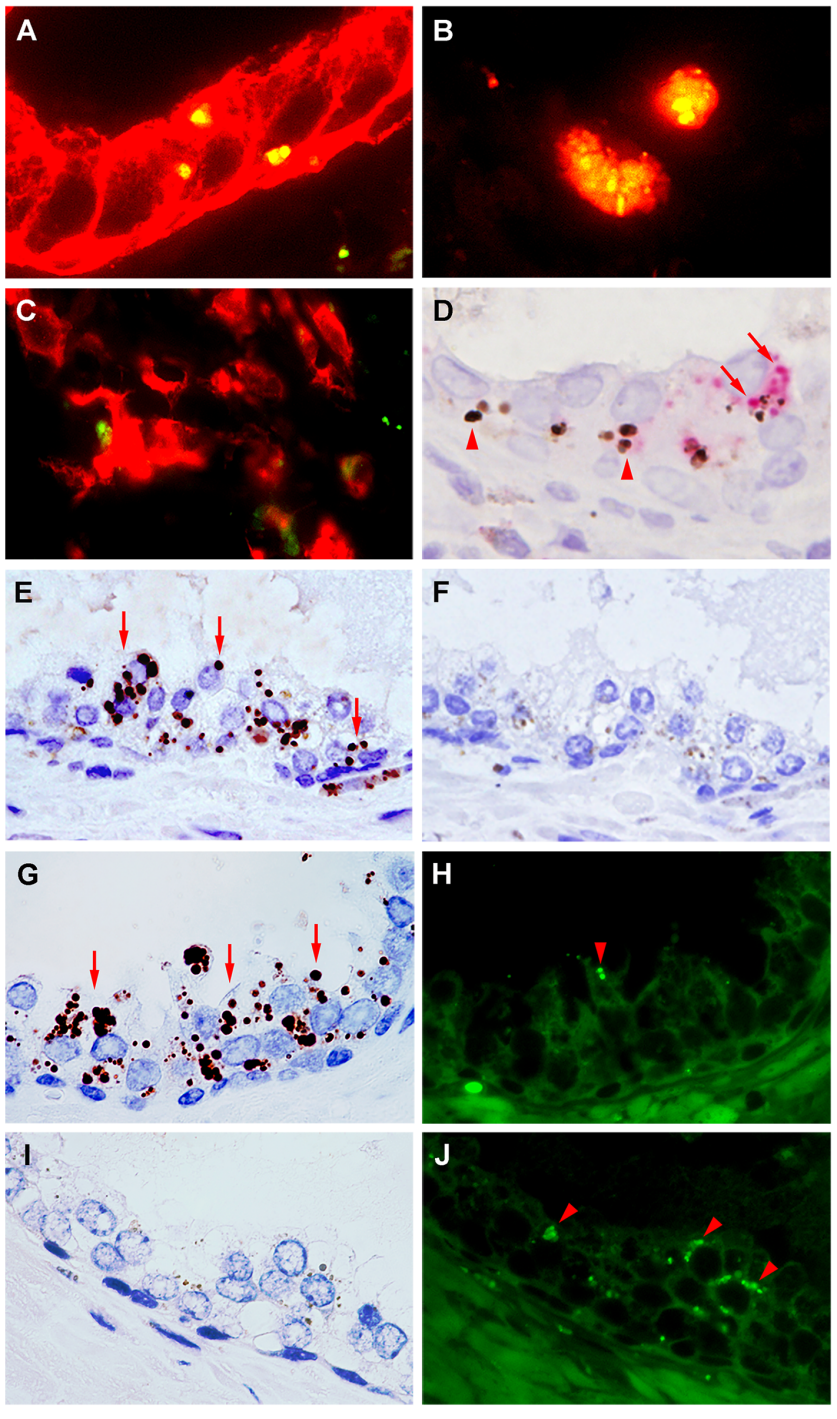
Characterization of *P. acnes*-positive cells and differentiation between *P. acnes*-positive signals and lipofuscin in prostate. Immunofluorescence double-staining of the *P. acnes*-positive cells in prostate tissues (A–C). Green signal (FITC): PAL antibody; red signals (TRITC): anti-cytokeratin antibody (A), anti-CD68 antibody (B), and anti-fascin antibody (C). Results of double-staining are merged in all pictures. In the prostate glands, all *P. acnes*-positive signals overlapped (yellow) with cytokeratin-positive glandular epithelial cells (A). In the prostate stroma, all *P. acnes*-positive signals overlapped (yellow) with CD68-positive macrophages (B) and did not overlap at all (green) with fascin-positive dendritic cells (C). Immunostaining with the PAL antibody followed by Fontana-Masson staining (D). Most of the red positive signals by PAL antibody (arrows) in the prostate glands did not overlap with the lipofuscin pigments (arrowheads), which are dark brown with Fontana-Masson staining. Immunoenzyme staining of prostate tissue with or without PAL antibody using serial prostate tissue sections (E, F). Many intracellular dark-brown signals (arrows) were observed in the section with the PAL antibody (E) but no such signals were observed in the section without the antibody (F). Identical part of the prostatic gland with or without immunoreactive signals by the enzyme method observed by both light and fluorescence microscopy (G–J). In the part of a prostatic gland with many positive antibody signals (arrows) (G), little lipofuscin auto-fluorescence (arrowhead) was observed in the identical area (H). In contrast, in the part of a prostatic gland with no positive antibody signals (I), a greater level of lipofuscin auto-fluorescence (arrowheads) was observed in the identical area (J).

Immunofluorescence double-staining confirmed that all of glandular signals detected by the PAL antibody were in the cytoplasm of the epithelial cells stained with anti-cytokeratin antibody (AE1/AE3) and all of the stromal signals detected by the PAL antibody were in macrophages stained with anti-CD68 antibody, but not with anti-fascin antibody (Figure 3A–C). Lipofuscin pigments were observed in some prostatic glands with intracellular *P. acnes*. These lipofuscin pigments were stained dark brown with Fontana-Masson and produced strong auto-fluorescence, and the PAL antibody did not cross-react with these lipofuscin pigments (Figure 3D–J).

**Figure 4:**
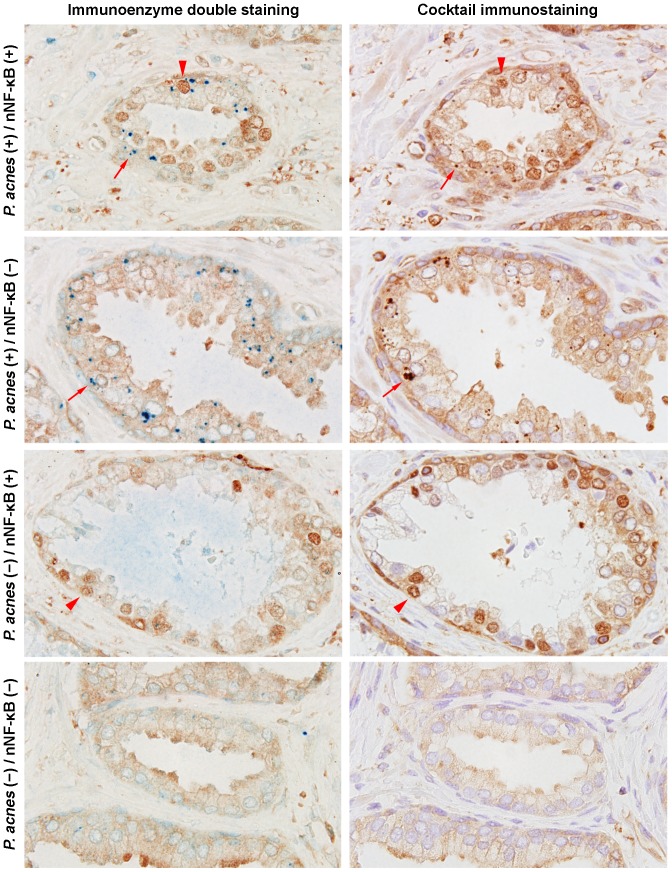
Comparison between immunoenzyme double-staining and cocktail immunostaining with the PAL antibody and anti-NF-κB antibody. In the double-staining (left column), the positive signals detected by the PAL antibody (arrows) and the positive nuclear signals by anti-NF-κB antibody (arrowheads) were visualized by blue and brown colors, respectively. In the cocktail staining (right column), both of the positive signals were visualized by the brown color. Representative glands that were categorized in each of four groups according to the status of intraepithelial *P. acnes* infection and nuclear NF-κB expression are shown in each row. The results of these parameters were the nearly same between immunoenzyme double-staining and cocktail immunostaining. nNF-κB: nuclear NF-κB.

**Figure 5:**
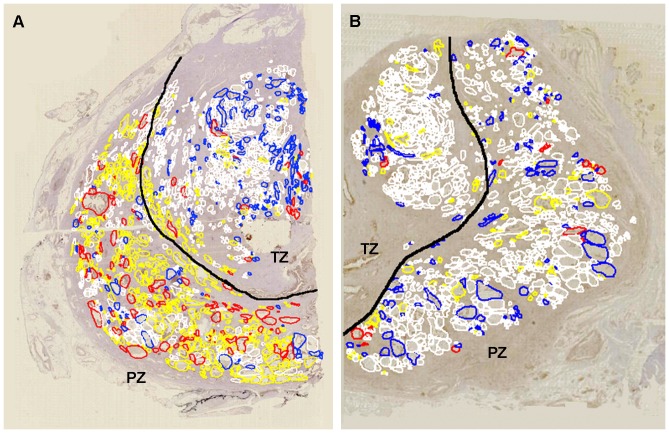
Distribution of glands with or without cytoplasmic *P. acnes* and/or nuclear NF-κB expression in prostate. Left, prostate cancer sample (A); right, control sample (B). Red circles, glands with both *P. acnes* and nuclear NF-κB expression; yellow circles, glands with *P. acnes* but no nuclear NF-κB expression; blue circles, glands with nuclear NF-κB expression but no *P. acnes*; and white circles, glands with neither *P. acnes* nor nuclear NF-κB expression. Black lines indicate the borderline between the peripheral zone (PZ) and transitional zone (TZ). Total number of glands examined by the virtual slide analyzer was 1894 in the sample (A) from a prostate cancer patient and 1465 in the sample (B) from a patient with bladder cancer but no prostate cancer. Note that more red and yellow circles were observed in the PZ area of the prostate cancer sample compared to both the PZ and TZ areas of the control sample as well as to the TZ area of the prostate cancer sample.

We also confirmed that the detection results of cytoplasmic *P. acnes* and nuclear NF-κB expression in prostatic glandular epithelial cells were almost the same between the immunoenzyme double-staining and the cocktail immunostaining that were used for analysis of the virtual slides (Figure 4). Simultaneous evaluation of *P. acnes* and NF-κB status in identical sections by cocktail immunostaining revealed a panoramic distribution of glands categorized into four groups on the virtual slides (Figure 5).

Prostate cancer cells in most cases were negative for the PAL antibody, with three exceptional cases (Figure 2G–H). The occupying area of cancerous glands with *P. acnes* infection in the whole area of the cancer lesion in the section was 5% in one case and less than 1% in the other two cases. PAL-positive stromal macrophages were detected in 13 (46%) of 28 cancer tissues.

### Detection frequency of *P. acnes* in prostate

Intraepithelial *P. acnes* of the non-cancerous prostate glands was detected in all of the samples, and stromal macrophages with the bacterium were observed in 27 of 28 cancer samples and 14 of 18 control samples.

**Figure 6:**
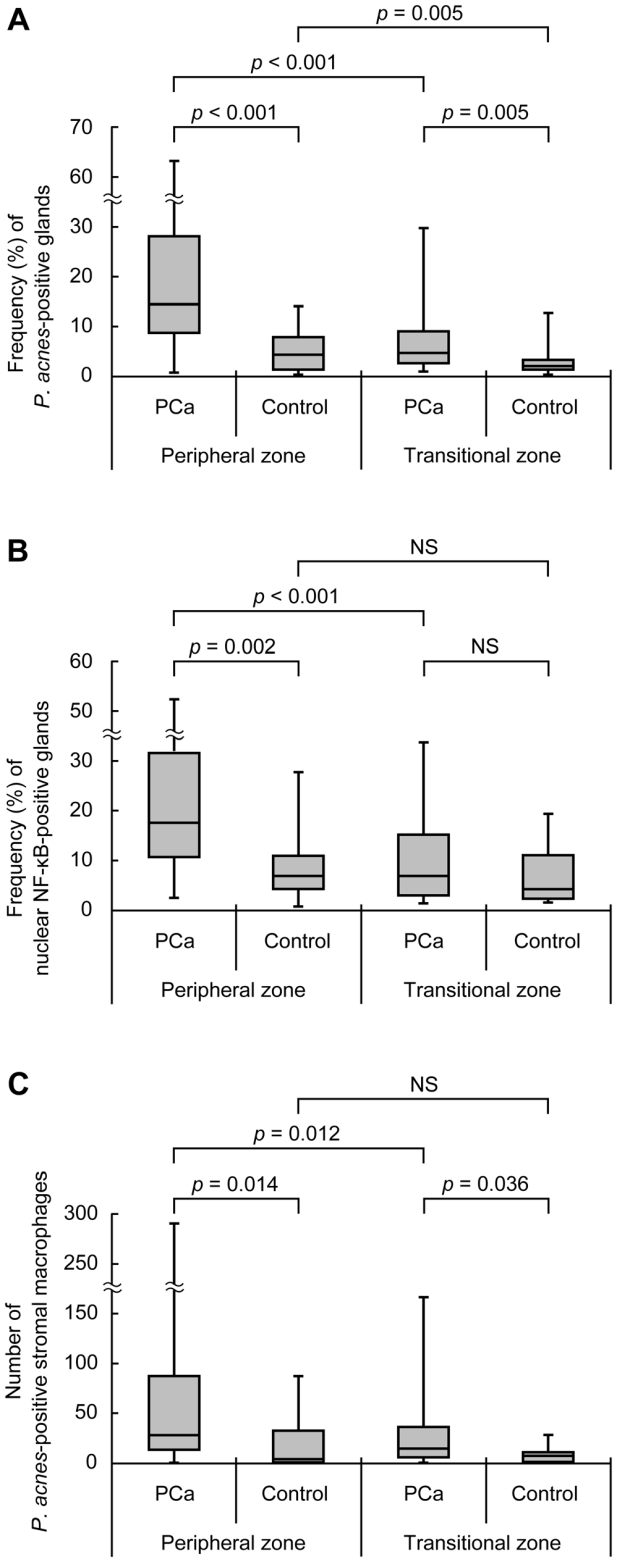
Detection results of *P. acnes* and nuclear NF-κB expression in prostate. Frequency (%) of *P. acnes*-positive glands (A) or nuclear NF-κB-positive glands (B), and the total number of *P. acnes*-positive stromal macrophages (C) in the peripheral and transitional zones of prostate cancer (PCa) and control samples. *P*-values were adjusted for multiple comparisons (4 comparisons in each panel) by Holm's method. NS: not significant.

The median [25^th^ and 75^th^ percentiles] frequency (%) of prostate glands with intraepithelial *P. acnes* was 14.4 [8.8, 28.0] in the PZ area and 4.9 [2.4, 9.1] in the TZ area of cancer samples (Figure 6A). In control samples, the frequency was 4.3 [1.3, 8.1] in the PZ area and 1.6 [1.1, 3.2] in the TZ area. The frequency was significantly higher in cancer samples than in control samples in both PZ and TZ areas. In both cancer and control samples, the frequency was significantly higher in the PZ area than in the TZ area.

The median number of stromal macrophages with *P. acnes* was 30 [10, 82] in the PZ area and 12 [3,35] in the TZ area of cancer samples, whereas the median number was 2 [0, 32] and 5 [0, 7] in control samples, respectively (Figure 6C). The number of stromal macrophages was significantly higher in the cancer samples than in the control samples in both the PZ and TZ areas. In cancer samples, the number was significantly higher in the PZ area than in the TZ area. The numbers of *P. acnes*-positive stromal macrophages correlated with the frequencies of *P. acnes*-positive glands in the PZ area of cancer samples (Spearman's rank correlation coefficient = 0.47, *p* = 0.015).

### Detection frequency of nuclear NF-κB expression

Nuclear NF-κB expression of glandular epithelial cells was detected in all samples. In the cancer samples, the median frequency (%) of NF-κB-positive glands was 17.1 [10.3, 31.4] in the PZ area and 7.1 [2.8, 14.8] in the TZ area (Figure 6B). In control samples, the frequency was 7.3 [4.4, 11.1] in the PZ area and 4.4 [2.1, 11.3] in the TZ area. In the PZ area, the frequency was significantly higher in cancer samples than in control samples. In cancer samples, the frequency was significantly higher in the PZ area than in the TZ area.

### Correlation between *P. acnes* infection and nuclear NF-κB expression

**Figure 7:**
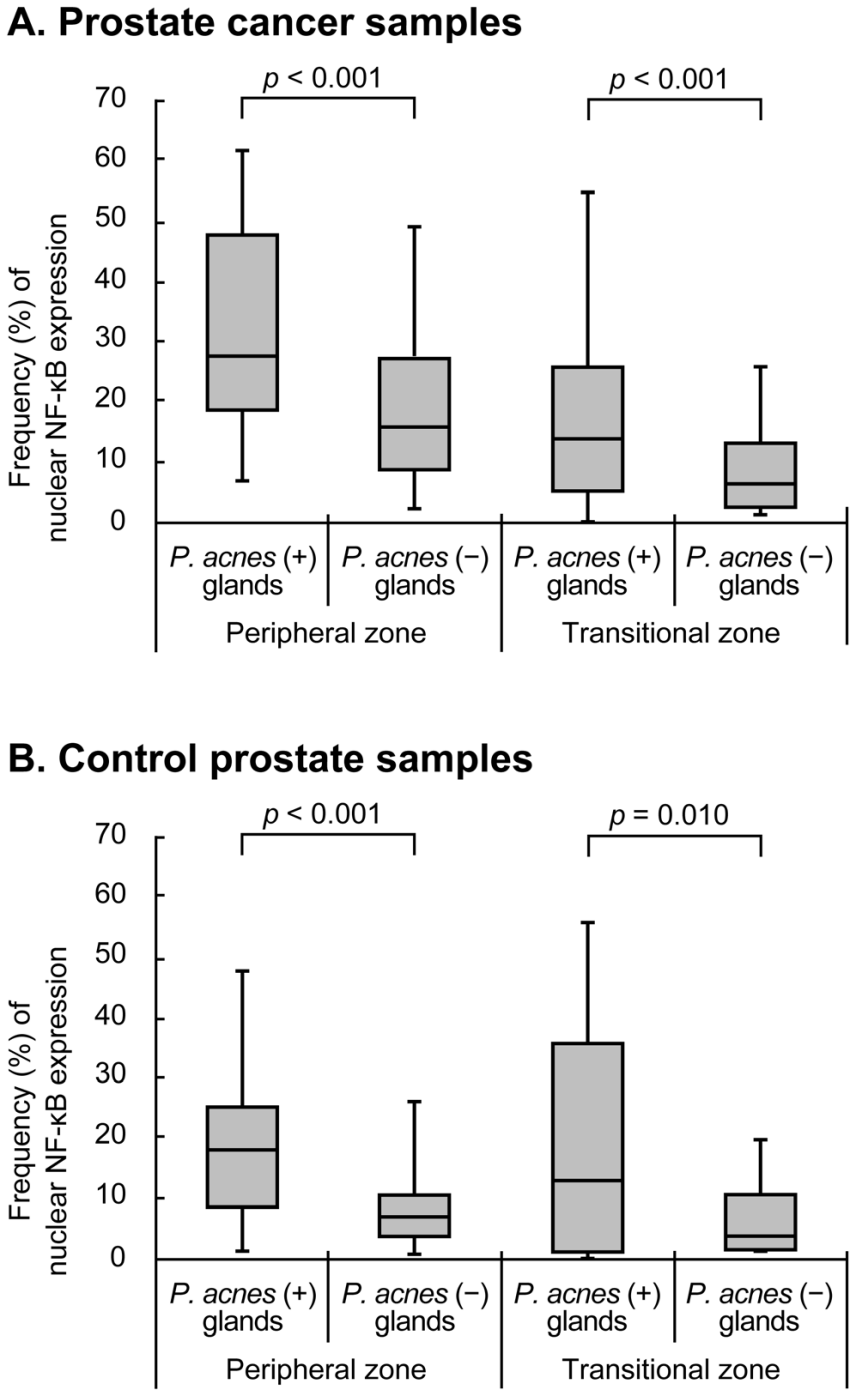
Comparison of nuclear NF-κB expression in the glands with or without cytoplasmic *P. acnes*. Frequency (%) of nuclear NF-κB expression in the glands with or without cytoplasmic *P. acnes* in the peripheral or transitional zones of prostate cancer (A) and control prostate (B) samples. *P*-values were adjusted for multiple comparisons (2 comparisons in each panel) by Holm's method.

In cancer samples, the median frequency (%) of nuclear NF-κB expression of the glands with *P. acnes* was 27.6 [18.3, 47.5] in the PZ area and 14.0 [5.8, 25.6] in the TZ area, whereas median frequency of the glands with nuclear NF-κB expression but without *P. acnes* was 15.9 [9.0, 27.2] in the PZ area and 6.7 [2.7, 13.7] in the TZ area (Figure 7A). In control samples, the frequency in *P. acnes*-positive glands was 17.8 [8.9, 24.8] in the PZ area and 13.0 [1.3, 36.1] in the TZ area, whereas the frequency in *P. acnes*-negative glands was 7.2 [4.1, 10.9] in PZ area and 4.1 [2.0, 10.7] in the TZ area (Figure 7B). The frequencies of nuclear NF-κB expression were significantly higher in *P. acnes*-positive glands than in *P. acnes*-negative glands in the PZ and TZ areas of both cancer and control samples.

### Correlation between inflammation and *P. acnes* infection status or NF-κB activation

**Figure 8:**
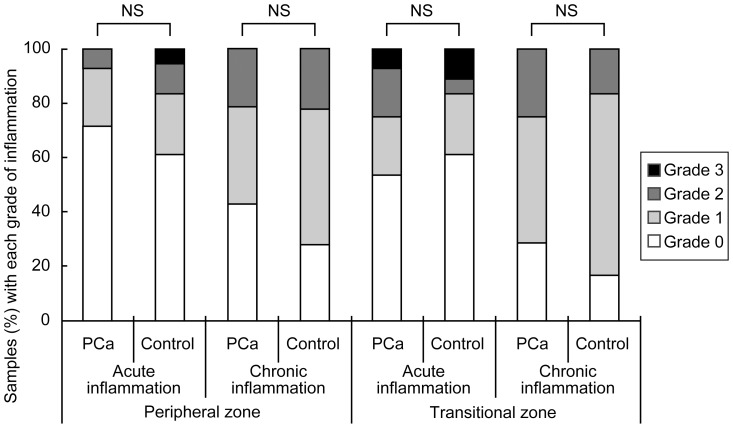
Grade of inflammation in peripheral and transitional zone of prostate cancer (PCa) and control samples. *P*-values were adjusted for multiple comparisons (4 comparisons in the panel) by Holm's method. NS: not significant.

Grade of acute or chronic inflammation in the PZ or TZ areas of the cancer and control samples is shown in Figure 8. In both PZ and TZ areas, the grade of inflammation did not differ significantly between the cancer and control samples. The grade of acute or chronic inflammation did not correlate with either the frequency of glands with cytoplasmic *P. acnes* or frequency of glands with nuclear NF-κB expression. The number of stromal macrophages with the bacterium correlated with the grade of chronic inflammation in both the PZ and TZ areas (Spearman's rank correlation coefficient = 0.44 and 0.61, *p* = 0.037 and 0.003, respectively) and with the grade of acute inflammation in the TZ area (Spearman's rank correlation coefficient = 0.53, *p* = 0.012).

## Discussion

Enzyme immunohistochemistry with a novel *P.acnes*-specific monoclonal antibody (PAL antibody) enabled us to visualize prostatic *P.acnes* under a light microscope for histopathologic analysis. In the present study, cocktail immunostaining with both the PAL antibody and anti-NF-κB antibody, followed by morphometric analysis of positive signals on virtual slides, revealed a possible correlation between intraepithelial *P. acnes* infection and NF-κB activation in prostate glands.

A diagnosis of prostatic *P. acnes* infection needs to be supported by histologic detection of the bacterium in tissue sections, because this indigenous bacterium may cause contamination and tissue invasiveness cannot be evaluated when traditional culture and polymerase chain reaction-based methods are applied [36]. In previous reports, prostatic *P. acnes* was visualized by fluorescence immunohistochemistry [31] or fluorescence in situ hybridization methods [14], although a precise histopathologic examination of the prostate lesions cannot be achieved by these methods. In the present study, we used the enzyme immunohistochemistry with the PAL antibody, which reacts with *P. acnes* with high specificity on routine histologic sections of the formalin-fixed paraffin-embedded prostate tissues. The PAL antibody detected the bacterium in all of the samples from both control and prostate cancer patients. The sensitivity of the antibody to detect *P. acnes* in prostate samples was high enough to detect this indigenous bacterium compared to those reported in previous studies, such as 82% with fluorescence immunohistochemistry [31], 50% with fluorescence in situ hybridization [14], and 35% with bacteria culture [13].

We previously constructed a similar monoclonal antibody (PAB antibody) to detect *P. acnes* in the lungs and lymph nodes [26], but the PAB antibody was not used for the present study because the antibody cross-reacts with lipofuscin pigments in prostate sections. In the present study, we successfully developed the PAL antibody to detect *P. acnes* without cross-reacting with lipofuscin pigments in prostate tissue samples. The PAL antibody used in the present study reacted with serotype I *P. acnes*, but not with serotype II *P. acnes*, whereas PAB antibody reacts with both serotype I and II *P. acnes* [26]. The serotype restriction of the PAL antibody may be associated with its high specificity to the epitope structure of *P. acnes* lipoteichoic acid, with which both PAB and PAL antibodies react.

The serotype restriction of the PAL antibody seems inconvenient for the purposes of the present study because both serotype I and II *P. acnes* have been isolated from prostates [29]. Thus, the results obtained here are only concerned with the infection status of serotype I *P. acnes* and no information was available concerning the infection status of serotype II *P. acnes*. As the invasiveness of this bacterium into epithelial cells is observed in 70% of serotype I isolates but not in serotype II isolates [29], however, the intraepithelial infection status of *P. acnes* obtained in the present study might not differ much from that obtained with the PAB antibody, which reacts with both serotype I and II *P. acnes*.

*P. acnes* was observed in the cytoplasm of some glandular epithelial cells of prostates from cancer and control patients. The presence of intraepithelial *P. acnes* of prostate glands with no histologic evidence of inflammatory reaction suggests that this indigenous bacterium may cause latent infection and persist in prostate glandular epithelium.

Tanabe et al. previously reported that intraepithelial infection of invasive serotype I *P. acnes* activates NF-κB in both a NOD1- and NOD2-dependent manner [30]. Generally, immunohistochemical detection of nuclear NF-κB expression in the cells indicates that NF-κB has been activated in the cell apart from the cause of its activation [34], [35]. In the present study, nuclear NF-κB expression was detected significantly more frequently in the *P. acnes*-infected glands than in non-infected glands. Furthermore, in the prostate cancer samples, the frequency of nuclear NF-κB expression was more prominent in the PZ glands than TZ glands, presumably associated with the predominant *P. acnes* infection to the PZ glands. These findings suggest that intraepithelial infection of *P. acnes* contributes to increasing the frequency of NF-κB activation of prostate glandular cells. *P. acnes*-induced intraepithelial NF-κB activation might have an important role in inflammation and carcinogenesis in the prostate.

*P. acnes* was also found in stromal macrophages of prostates from cancer and control patients. Many or a few small round bodies were found in the cytoplasm of stromal macrophages accumulating in the foci of inflammation and the total number of *P. acnes*-positive macrophages correlated with the grade of chronic inflammation. These *P. acnes*-positive macrophages were also sometimes observed in prostatic glands and their luminal spaces. These findings suggest that some prostatic inflammation might be caused by this indigenous bacterium. Furthermore, the lack of a significant correlation between the grades of inflammation and the *P. acnes* or NF-κB status of glandular cells may reflect multiple causes of prostate inflammation, such as infectious agents other than *P. acnes*, dietary habits, and hormonal changes [2], although Cohen et al. reported that a significantly higher degree of prostatic inflammation is observed in cases positive for *P. acnes* by bacterial culture [13].

Although the infection route of *P. acnes* to the prostate is unknown, frequent isolation of *P. acnes* from urine samples suggests the possible entry of *P. acnes* into the prostate through the urethra [37]. Recently, a mouse model of chronic prostatic inflammation was established using transurethral catheterization of *P. acnes* [38], and intraepithelial bacteria were found in mouse prostate glands using immunohistochemistry and in situ hybridization methods. Thus, the intraepithelial *P. acnes* of human prostate glands found in our study might have been caused by latent *P. acnes* infection due to continuous exposure for a certain period to the indigenous bacterium via the ascending urinary route. Latent intraepithelial *P. acnes* infection can be activated under certain host or environmental conditions [27], and may have caused some of the prostatic inflammation. Macrophages with *P. acnes* observed in the study seem to have phagocytosed the bacterium in the inflammatory state caused by *P. acnes* proliferation in the prostatic stromal and glandular luminal spaces. Prostatic *P. acnes* may also contribute to the development of prostate cancer due to persistent chronic inflammation caused by this low-virulence indigenous bacterium.

In the present study, we examined non-cancerous areas of prostates from control and prostate cancer patients and focused mainly on the status of *P. acnes* infection in non-cancerous glandular epithelial cells. Although most cancer cells in the cancerous prostate glands showed no positive signals, there were some exceptional cases. In 3 (11%) of 28 samples with prostate cancer, some clustered cancer cells had the same intracellular signals detected by the PAL antibody as those found in non-cancerous glands. Because *P. acnes* infection can also occur in cancer cells, as shown in previous studies [29], [30], infection of cancer cells may have incidentally occurred after the cancer developed and settled in the area. The frequency of *P. acnes* infection in the cancerous glands was far lower than that in non-cancerous glands, presumably because of a shorter period of exposure to indigenous *P. acnes* in the case of cancerous glands.

In the present study, the frequencies of *P. acnes*-positive glands and nuclear NF-κB-positive glands and the number of *P. acnes*-positive stromal macrophages were significantly higher in cancer samples than control samples. Furthermore, in cancer samples, these parameters for *P. acnes* infection were higher in the PZ area where most prostate cancers are located, compared to those in the TZ area. The frequent detection of prostate glands with intraepithelial *P. acnes* infection and NF-κB activation in the PZ area of cancer samples suggests a possible association between *P. acnes* infection and prostatic carcinogenesis.

## Conclusions

We developed a novel anti-*P. acnes* monoclonal antibody that can detect *P. acnes* without cross-reacting with lipofuscin pigments in formalin-fixed paraffin-embedded prostate tissue samples. Immunohistochemical analysis of radical prostatectomy samples with or without prostate cancer using this novel antibody revealed the bacterium within some non-cancerous glandular epithelium and stromal macrophages that were most frequently found in the PZ area of prostate cancer samples. Intraepithelial *P. acnes* infection in non-cancerous prostate glands and inflammation caused by the bacterium may contribute to the development of prostate cancer.
